# Essential Oils: Pharmaceutical Applications and Encapsulation Strategies into Lipid-Based Delivery Systems

**DOI:** 10.3390/pharmaceutics13030327

**Published:** 2021-03-03

**Authors:** Cinzia Cimino, Oriana Maria Maurel, Teresa Musumeci, Angela Bonaccorso, Filippo Drago, Eliana Maria Barbosa Souto, Rosario Pignatello, Claudia Carbone

**Affiliations:** 1 Laboratory of Drug Delivery Technology, Department of Drug and Health Sciences, University of Catania, Viale A. Doria 6, 95125 Catania, Italy; cinzia.cimino@phd.unict.it (C.C.); teresa.musumeci@unict.it (T.M.); angela.bonaccorso@unict.it (A.B.); rosario.pignatello@unict.it (R.P.); 2 Department of Biomedical and Biotechnological Sciences, University of Catania, 95125 Catania, Italy; orianamaurel@hotmail.it (O.M.M.); filippo.drago@unict.it (F.D.); 3 Department of Pharmaceutical Technology, Faculty of Pharmacy, University of Coimbra, 3000-548 Coimbra, Portugal; ebsouto@ff.uc.pt; 4 CEB—Centre of Biological Engineering, Campus de Gualtar, University of Minho, 4710-057 Braga, Portugal

**Keywords:** extraction method, anti-inflammatory, antioxidant, antimicrobial, wound healing, anxiolytic, nanoemulsions, microemulsions, liposomes, SLN, NLC

## Abstract

Essential oils are being studied for more than 60 years, but a growing interest has emerged in the recent decades due to a desire for a rediscovery of natural remedies. Essential oils are known for millennia and, already in prehistoric times, they were used for medicinal and ritual purposes due to their therapeutic properties. Using a variety of methods refined over the centuries, essential oils are extracted from plant raw materials: the choice of the extraction method is decisive, since it determines the type, quantity, and stereochemical structure of the essential oil molecules. To these components belong all properties that make essential oils so interesting for pharmaceutical uses; the most investigated ones are antioxidant, anti-inflammatory, antimicrobial, wound-healing, and anxiolytic activities. However, the main limitations to their use are their hydrophobicity, instability, high volatility, and risk of toxicity. A successful strategy to overcome these limitations is the encapsulation within delivery systems, which enable the increase of essential oils bioavailability and improve their chemical stability, while reducing their volatility and toxicity. Among all the suitable platforms, our review focused on the lipid-based ones, in particular micro- and nanoemulsions, liposomes, solid lipid nanoparticles, and nanostructured lipid carriers.

## 1. History of Essential Oils

Essential oils (EOs) have been known for millennia: during the Neolithic Age (before 4000 B.C.) they were extracted from plants by simple squeezing and this was the turning point that gave rise to the sedentary lifestyle. This led to the construction of the first sacred monuments where EOs were used in rituals. Besides clay cuneiform writing pieces (2600 B.C.) discovered in Mesopotamia, one of the oldest discoveries that proves the use of EOs for medical purposes is an Egyptian papyrus dated 2551–2528 B.C. [1]. Moreover, the Egyptians used aromatic oils for beauty treatments and spiritual rituals, including the ceremony of mummification; the use of EOs has allowed a good preservation of some mummies. Generally, the Egyptians used to extract oils by infusion of the plant in a fatty substance and consequent boiling, in this way, the aroma evaporated and fixed itself in the fat [1]. Based on some evidences, they were aware of an oil extraction procedure by distillation, even if this was not commonly used. “Shennong’s Herbal” is the oldest medical book, dating back to 2700 B.C. and belonging to the Chinese tradition: it contains the usage guidelines for 365 plants. China is still the world’s leading producer of EOs. The use of EOs has been a fundamental part of Indian medical practice for five thousand years. Ayurveda is a traditional book that describes the use, both medical and religious, of over 700 different types of plants, and it is still the basis of the Indian healthcare [1]. The discovery of the stimulating and calming properties of some EOs belongs to the Greek culture; in the temples of Asclepius and Aphrodite, marble tablets with engraved recipes of some medicinal aromas have been found [2]. Hippocrates, considered the father of modern medicine, documented the medicinal importance of over 300 plants [3]. He strongly believed in the medical benefits of fumigation with aromatic oils in the treatment of the plague; he was also convinced that the topical use of aromatic preparations could produce systemic effects. Romans, under the influence of Greeks, used EOs for bathing and massages. Among the findings belonging to Romans, there is an interesting treatise on herbal medicine dating back to the first century A.D., De Materia Medica, in which Pedanius Dioscorides reported more than 600 remedies [1]; in the following centuries the effectiveness of most of them was confirmed. Not less than 12 different types of EOs are mentioned in the Holy Bible, both in Old and New Testament [3]. During the Middle Ages, the Catholic Church banned the use of EOs for medical treatment: those who used them were burned at the stake, because they were associated to witchcraft. Despite the persecutions, the monks secretly kept the tradition [1]. A writing found in the island of Cyprus and dating back to the Ottoman Empire (1571–1878) describes 231 plants and 494 prescriptions of herbs, which were used by Orthodox monks in their hospitals [4]. In the 10th century, the Persian alchemist Avicenna gave a great contribution to science and medicine, discovering the distillation method: the first EO extracted was from rose [1]. During the 13th century, there was a great expansion of the practice of oil distillation, extracting a great variety of aromas. In the 17th century, the use of natural extracts was abandoned due to the discovery of chemical substances. The interest in EOs was rekindled in 1928 thanks to Rene-Maurice Gattefossé, who coined the term “Aromatheraphie” and discovered the healing properties of lavender EO. In 1990, the book “L’Aromatherapie Exactement” was published, in which Daniel Pénoël and Pierre Franchomme reported the medical properties of more than 270 EOs, and this was the starting point for many studies.

## 2. Principal Extraction Methods for Essential Oils

**Figure 1:**
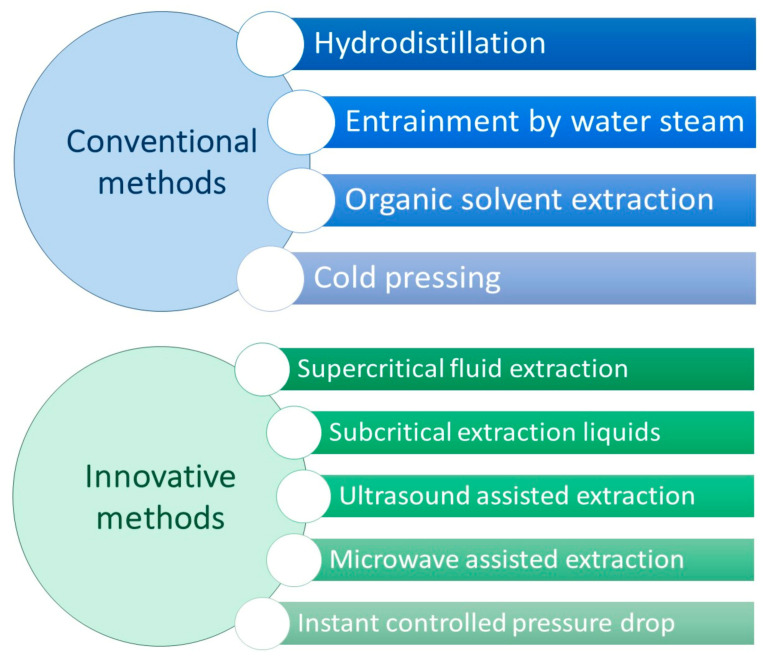
Schematic summary of conventional and innovative extraction methods.

According to the definition given by the International Organization for Standardization (ISO), the term EO is related to a “product obtained from vegetable raw material, either by distillation with water or steam, or from the epicarp of citrus fruits by a mechanical process or by dry distillation.” In order to extract EOs from vegetable raw material, different methods can be exploited, which can be classified in two categories: the classical (or conventional) methods and the innovative (or advanced) ones [5]. The choice of the extraction method is decisive, since it determines the type, quantity, and stereochemical structure of the essential oil molecules [6]. It is important to underline that the oil composition is strictly influenced by the variety of the plant, the place where it has grown (climate and environment, any stress suffered), the type of nutrition and fertilizers used [7]. Below a brief description of the main methods is provided. Conventional methods (Figure 1) are not very advantageous due to the thermolability of EOs (high temperatures used) and the low quality of the extract (production of chemical artefacts, due to long extraction time). Among these methods *Hydrodistillation* can be considered a simple and old technique (it was used by Avicenna in 10th century). The parts of the plants (particularly flowers and petals) are directly boiled inside the water; this alembic is connected with a condenser (which holds the essential oil) and a decanter (that collects the evaporated water) [8]. Another technique used for EOs extraction is *Entrainment by water steam*. The principle is the same as hydrodistillation, but the plant is not in contact with water. There are several variants of this method, such as vapor-hydrodistillation, steam distillation, hydrodiffusion [9]. *Organic solvent extraction* is the third common method. The parts of the plant are put inside an organic solvent and macerated; the solvent is removed under pressure, in order to concentrate the extract. EOs extraction can also be performed by *cold pressing*. This is a traditional method used to extract EOs from citrus fruit zest. Oil sacs or oil glands break during extraction, releasing the volatile oil, which is mechanically recovered by centrifugation [10].

Innovative methods (Figure 1), compared to the classical ones, have numerous advantages, due to the reduction in the use of solvents and in the extraction time. *Supercritical fluid extraction* is one of the most common innovative methods. The solvent used is generally CO_2_, which is compressed and heated to reach the supercritical state; passing through the parts of the plant, supercritical CO_2_ is loaded by volatile components and extracts; in the end, the depression phase allows the separation of the oil [11]. *Subcritical extraction liquids* can be exploited using water and CO_2_ at the subcritical state, thus using lower temperatures. In *ultrasound-assisted extraction*, the vegetable is immersed in water or other solvent: the ultrasound action favors the release of EOs from the plant, owing to mechanical vibration of the cellular walls and membranes [12]. *Microwave-assisted extraction* is one of the most potent and promising extraction methods. There are many variants: solvent free microwaves extraction (SFME); microwave hydrodiffusion and gravity (MHG); microwave steam distillation (MSD); microwave steam diffusion (MSDf). Farhat and co-worked studied the use of MSDf to extract *Lavandula* EO [13]. Comparing with other extraction methods of *Lavandula* EOs proposed by Périno-Issartier and co-workers, it was proved that MSDf is more efficient in terms of extraction time, and the quality of the extract is superior [14]. *Instant controlled pressure drop* (DIC) method is composed of four steps: first, the plants have to be placed under the vacuum, the steam bath is applied at determined pressure and temperature, then the void is instantly detained and the sample is taken back to the atmospheric pressure.

In Table 1 the advantages and drawbacks of the different EOs extraction methods described are reported.

## 3. Essential Oils Main Applications and Limits in the Pharmaceutical Field

EOs are liquid mixtures of volatile components produced by aromatic plant secondary metabolism. The main components are monoterpenes and sesquiterpenes and, to a lesser extent, aromatic and aliphatic compounds, therefore, the complex mixtures of compounds usually include terpenoids, alcohols, ethers, esters, ketones, and aldehydes in variable concentrations, and can be obtained by over 60 families of plants [34]. Concentration variability leads to significant heterogeneity in the products used in different studies and makes a comparison among results difficult [35]. Generally, EOs contain about 20–60 components up to more than 100 single substances, at quite different concentrations. Usually, two or three are the major components at high concentrations (20–70%) compared to other components present in trace amounts [6]. EOs show characteristic physical properties (e.g., color and solubility in water) that can represent some criteria for the classification and qualification to different groups [36]. The use of EOs is limited due to the following drawbacks: hydrophobicity (and, consequently, insolubility in water); instability (caused by hydrolysis and oxidation); high volatility [37,38]. Therefore, nowadays the researchers’ efforts are focused on the possibility to use innovative formulation for the EO encapsulation. However, the therapeutic application of EOs as pure compounds was studied, mainly for external applications (mouthwashes or inhalation), since it is generally safe, except for some skin reactions and UV sensitivity (the exposure to sunlight could cause irritation or darkening of the skin). It is important to not exceed in the amount of the administered dose of EO and to avoid their application on broken skin, which would induce the occurrence of significant systemic absorption and, therefore, serious side effects. The administration of EOs by inhalation is the most rapid way (followed by the topic one). The use of strong oils (for direct inhalation or for diffusing) is not recommended because they may cause eye irritation. EOs oral administration is rare, even if it is considered safe, and the EOs are generally diluted in olive oil or milk.

### 3.1. Anti-Inflamamtory and Antioxidant Activities

Inflammation is an essential biological mechanism, which provides a response to harmful stimuli coming from the outside. The inflammation can be acute, when the stimulus produces a limited response over time, and therefore ends within a few days. It may also be chronic, if the external insult persists over time, the person is particularly sensitive or suffers from autoimmune pathologies. Acute inflammation is characterized by five symptoms: *calor* (increase in local vascularization with elevation of the temperature), *tumor* (formation of an exudate, which causes oedema), *rubor* (active hyperaemia causing redness), *dolor* (the inflammatory components of exudate cause stimulation of sensorial nerves and compression), and *functio laesa* (impairment of the functionality of the inflamed tissue). Chronic inflammation is mainly caused by the migration, into tissues, of mononuclear blood cells, such as monocytes and lymphocytes. The most effective therapy is the etiological one, acting on the cause of the inflammation, for example by administering an antibiotic if the cause is an infection. If this is not enough, or if the cause is not clear, traditional anti-inflammatory drugs are also used. They belong to two categories: non-steroidal anti-inflammatory drugs (NSAIDs) and corticosteroids. Especially when treating chronic inflammation, prolonged intake of these drugs could produce significant adverse effects, such as ulcers and gastritis for NSAIDs, or the risk of over-infection using glucocorticoids. For this reason, there is now a growing interest in the use of natural compounds as adjuvants for these therapies, especially chronic ones. The importance of natural medicines in the treatment of many inflammatory diseases has always been well-known. The “water of the Queen of Hungary”, an infusion of rosemary EO, dates back to the 16th century, as Queen Isabell used it to cure rheumatism; it was at the court of Louis XIV that this preparation spread in the form of medicine [39]. Many other historical evidences, linked to traditional medicine, indicate the use of rosemary EO to treat inflammatory conditions of different nature, like rheumatoid arthritis, asthma, bronchitis, etc., [39,40,41]. One of the most commonly used in vivo model to analyze the anti-inflammatory power of EOs is the carrageenan-induced pleurisy experiment: a group of mice is pre-treated with the EO, while the others are monitored as positive and negative control; each mouse is treated with the inflammatory agent, which in this situation is carrageenan, and, at defined intervals, the paw oedema is measured. Using the above mentioned model, Ogunwande et al. analyzed *Bougainvillea glabra* EO to assess its effectiveness as an anti-inflammatory agent [42]. The groups of mice were treated with different concentrations of the EO and then compared with the standard group (treated with diclofenac) and the control group (administered with saline solution). Anti-inflammatory activity of the EO was demonstrated, and it was related to its ability of inhibiting both histamine- and prostaglandins-mediated responses. Besides carrageenan, it is also possible to use dextran as an inflammatory agent, as operated by Rodrigues and co-workers who exploited both experiments to analyze *Ocimum basilicum* EO [43]. In the same work, also histamine and arachidonic acid were used as a model to analyze their inflammatory pathways. All doses administered, even lower ones, showed excellent anti-inflammatory properties in all the performed experiments. Also, *Cinnamomum osmophloeum* Kanehira leaf EO demonstrated anti-inflammatory activity, due to the presence of the two main components, cinnamaldehyde and linalool. In another study, some mice were pre-treated with one of the two compounds and then all were injected with endotoxin to activate the inflammatory pathway [44]. The results obtained from these analyses were consistent and extremely detailed: in mesenteric lymph nodes and spleen, pre-treated mice have developed fewer inflammation mediators than the non-treated ones, including interleukin (IL)-1β, tumor necrosis factor (TNF)-α, IL-18, interferon (IFN)-γ, nitrate/nitrite, etc. Furthermore, the expression of different genes, proteins, receptors, and enzymes, induced by endotoxin, was suppressed. *Artemisia argyi* EO was widely used in China for the treatment of chronic bronchitis, due to the anti-inflammatory activity of his components, as asserted by Chen et al. [45]. Among many others, they proved that the dose-dependent anti-inflammatory activity of *Artemisia argyi* EO is linked to the inhibition of two important events: the release of pro-inflammatory mediators, such as TNF-α, IL-6, IFN-β, NO, PGE2, etc., and the phosphorylation of JAK2 and STAT1/3. The suppression of JAK/STATs pathway also demonstrated the antioxidant property of this EO against reactive oxygen species (ROS). Upon this point, besides the anti-inflammatory properties of EOs, experienced for centuries, in a more contemporary age, the researchers’ interest has also focused on the antioxidant properties. Oxidation occurs when the balance between free radicals and antioxidants is shifted toward the oxidizing species. Free radicals could induce a plethora of biological events, affecting various cell’s components, like DNA or proteins, thus inducing several consequential pathologies, such as cancers, neurodegenerative diseases and chronic inflammation disorders. Certainly, environmental, dietary or lifestyle conditions could result in an increased amount of radical species produced by the organism and, therefore, in a condition of oxidative stress. It is well-known that through diet it is possible to compensate the inadequacy of endogenous antioxidants by consuming foods rich in vitamin A, C, and E, resveratrol, lycopene, and so on. The benefits of antioxidant supplementation are still subject of debate and further investigation, as an attempt is nowadays focused to quantify the extent of this improvement. Many EOs have antioxidant properties, and the use of EOs as natural antioxidants is a field of growing interest because some synthetic antioxidants such as butylated hydroxyanisole (BHA) and butylated hydroxytoluene (BHT) are now suspected to be potentially harmful to human health. Addition of EOs to edible products, either by direct mixing or in active packaging and edible coatings, may therefore represent a valid alternative to prevent autoxidation and to prolong shelf life. The evaluation of the antioxidant performance of EOs still represents a crucial issue, because many commonly used “tests” are inappropriate and give contradictory results that may mislead future research. The chemistry explaining EO antioxidant activity is discussed along with an analysis of the potential in food protection. Literature methods to assess EOs antioxidant performance are critically reviewed. Currently, antioxidants have become essential in nutrition because of their ability to protect the body against oxidative cell damage that can produce dangerous diseases. EOs are one of the major sources of different bioactive molecules that directly act on the body. Literature studies describe a variety of approaches to demonstrate the antioxidant power of EOs. Among the most common essays we can identify the DPPH (1,1-diphenyl-2-picrylhydrazyl radical) method: this radical is characterized by an absorption band at 515 nm, which changes after the administration of an antioxidant; the radical scavenger activity of a compound is often quantified as IC_50_, which is the amount of antioxidant necessary to halve the absorbance of the DPPH. This method was used by Silva and co-workers, who analyzed lavender EO, extracted from *Lavandula augustifolia* [46]. As an antioxidant, lavender EO showed good IC_50_ values, indicating that antioxidant activity is dose dependent, with a peak of activity associated with concentration of 150, 120, and 100 mg/mL. Da Silva also investigated the anti-inflammatory activity of lavender EO performing the carrageenan assay on mice, comparing it to saline solution as negative control and dexamethasone as positive control. The leukocytes diminishing effect was qualitatively similar to that produced by dexamethasone, which, however, proved to be more effective. The author suggested an involvement of a G protein-coupled receptor and/or phospholipase C/inositol phosphate messenger. Another species of lavender EO, *Lavandula x intermedia* “Sumian”, was studied by Carbone and co-workers [47] together with *Rosmarinus officinalis* L. and *Origanum vulgare* subsp. *hirtum* EO, to assess their antioxidant and anti-inflammatory activities. The 2,2-diphenyl-1-picrylhydrazyl (DPPH) analysis indicated that only *Origanum* possesses a remarkable antioxidant activity, with a value of 216 μg/mL of Trolox equivalents. This is certainly attributable to the presence of large amounts of phenolic compounds, which, on the other hand, are scarcely present in *Lavandula* and *Rosmarinus*. The same study also demonstrated the anti-inflammatory activity of the three EOs, in the following order: *Lavandula* > *Rosmarinus* ≥ *Origanum* [47]. Another widely used reagent is the 2,2-azino-bis-3-ethylbenzothiazoline-6-sulfonic acid (ABTS) cationic radical, whose protocol of use also consists in measuring the variation of the specific absorbance. It was used by Elaguel et al., in conjunction with the DPPH method and phosphomolybdenum assay, for demonstrating the antioxidant activity of *Lawsonia inermis* EO, thus suggesting the EO possible application as adjuvant in anti-cancer therapies [48]. DPPH and ABTS assays were also used to confirm the antioxidant property of the EO extracted from blossoms of *Citrus aurantium* L. var. *amara* Engl. (CAVAO), which has also shown an even more marked anti-inflammatory activity, that could be exploited for the development of functional foods to be used in patients with inflammatory diseases [49]. Another assay worth mentioning is the ferric-reducing ability of plasma (FRAP), which measures the ability to cause ferric reduction. Together with other assays, it was useful to analyze the main active components of *Anethum graveolens* seed EO, which were demonstrated to be carvone, limonene, and camphor, thus showing that carvone is an excellent antioxidant due to the presence of unsaturated hydroxyl group [50]. Furthermore, the antioxidant activity of *Mentha spicata* was confirmed by performing FRAP and ABTS assays, suggesting a possible use of this EO in the pharmaceutical field [51].

### 3.2. Antimicrobial Activity and Wound Healing

The treatment of microbial infections has always been one of the highest and most ambitious goals in the pharmacological field. The ability of microorganisms to continuously develop new drug resistance mechanisms makes this an ever-evolving field. In fact, the resistance toward antibiotics is one of the main impediments to perform an appropriate treatment for certain infections. Despite the availability of many classes of antibiotics, and therefore a large number of molecules, today this problem is increasingly relevant and alternative therapies, such as EOs, are being investigated to overcome it. Even Hippocrates, more than two thousand years ago, affirmed that the fumigation of EOs was useful to protect from the plague [3]. This was proved in the Middle Ages when some gangs of robbers, due to the assumption of tinctures containing EOs, were able to steal from the houses of the plague victims without being infected. For this reason, EOs have been extensively studied and have been considered as a valid alternative therapy against bacterial infections. The antibacterial activity of EOs depends on the presence of certain components, especially mono- and sesquiterpenes, which are known as efficient antimicrobial agents [52]. Moreover, the highest antibacterial activity belongs to phenolic groups, followed by cinnamic aldehydes. Other groups are also important, including alcohols, aldehydes, ketones, ethers, and hydrocarbons [53]; specifically, antimicrobial activity against Gram-positive bacteria is related to the amount of long-chain alcohols and aldehydes, as stated by Shojaee-Aliabadi [52], while antimicrobial activity of alcohols is directly proportional to their molecular weight [52,54]. Many EOs have been studied to determine their effectiveness as antibacterial, and the most commonly used assays are the diameter of the inhibition zone (DIZ) evaluation, by measuring the halo diameter, and the determination of the minimum inhibitory concentration (MIC) and/or minimum bactericidal concentration (MBC), through the microdilution method. The research conducted by Hammer et al. [55] demonstrated the antimicrobial activity of a large number of plant oils and extracts, derived from 37 genera, which were investigated against different kinds of microorganisms, such as *Acinetobacter baumanii*, *Aeromonas veronii biogroup sobria*, *Candida albicans*, *Enterococcus faecalis*, *Escherichia coli*, *Klebsiella pneumoniae*, or *Pseudomonas aeruginosa*. As a result, tested EOs and plant extracts have demonstrated in vitro antibacterial and antifungal activity against a wide range of organisms, comprising Gram-positive and Gram-negative bacteria and a yeast [55]. Particularly relevant were the results obtained by *Cymbopogon citratus*, *Origanum vulgare*, and *Pimenta racemosa* EOs. *Aniba rosaeodora*, *Coriandrum sativum*, *Cymbopogon martinii*, *Melaleuca alternifolia*, *Melaleuca quinquenervia*, *Mentha piperita*, *Mentha spicata*, *Salvia officinalis*, and *Origanum majorana* results were also noteworthy. The lowest MIC value (0.008% *v/v*) was measured by *Vetiveria zizanioides* against *S. aureus*, while *Thymus vulgaris* showed a great activity against *C. albicans* and *E. coli*, with a MIC value of 0.03% *v/v*. Moreover, the study carried out by Nikolić, Jovanović et al. [56] confirmed a significant antimicrobial activity of five EOs *Mentha piperita*, *Mentha pulegium*, *Lavandula angustifolia*, *Satureja montana*, and *Salvia lavandulifolia*. Seven bacterial species—namely *Streptococcus mutans*, *Streptococcus sanguis*, *Streptococcus salivarius*, *Streptococcus pyogenes*, *Pseudomonas aeruginosa*, *Lactobacillus acidophilus*, and *Enterecoccus faecalis*—as well as fifty-eight clinical oral *Candida* spp. were subjected to the treatment with these EOs, confirming their potentiality as antibacterial agent [56]. The antimicrobial potential of the tested EOs increased in the order: *L. angustifolia* < *M. pulegium* < *S. lavandulifolia* < *M. piperita* < *S. montana*. *P. aeruginosa* [57]. As extensively reported by Bilia et al. [58], *Artemisia annua* has showed great antimicrobial activity against Gram-positive and Gram-negative bacteria, and also fungi. The review deeply describes the interesting studies on different Gram-positive bacterial strains, with the lowest MIC values reported for *Staphylococcus aureus*, *Enterococcus hirae*, *Enterococcus faecalis*, *Bacillus cereus*, *Bacillus subtilis*, *Bacillus thuringiensis*, and *Bacillus* spp. (all ranging from 0.00781 mg/mL of *B. subtilis* to 0.053 mg/mL of *B. cereus*). Gram-negative lowest MIC values were 0.013 mg/mL of *Escherichia coli*, 0.026 mg/mL of UPEC (*Escherichia coli* Uropathogenic), and 0.025 mg/mL of *P. aeruginosa*. Furthermore, the most susceptible fungi were described to be *Candida albicans* and *Saccharomyces cerevisiae*, with a 2 mg/mL MIC value. The interesting antimicrobial property of *Artemisia annua* is strictly related to all its components as a whole. In fact, the same research group further analyzed the antimicrobial activity of its main components (artemisia ketone, 1,8-cineole and camphor), in comparison with the whole EO [59]. Disk diffusion method and broth microdilution assay were performed on the most common food pathogens, namely *Escherichia coli*, *Salmonella enteritidis*, *Salmonella typhi*, *Yersinia enterocolitica*, and *Listeria monocytogenes*. As expected, the components separately tested were not able to perform as well as the entire EO, even if the values of the diameters of inhibition zones and the obtained MBC values were remarkable, thus suggesting that the synergism and/or antagonism occurring between the components is necessary to achieve such a great antimicrobial activity. Zhang et al. analyzed the antimicrobial activity of *Melaleuca alternifolia* EO in different strains: *Escherichia coli*, *Staphylococcus aureus*, *Pseudomonas aeruginosa*, *Penicillium italicum* Wehmer, and *Penicillium digitatum* Sacc. [60]. The EO showed a good antimicrobial activity toward all the tested strains, with a slightly more pronounced action toward Gram-positive bacteria, compared to Gram-negative bacteria and fungi. This is not surprising since it is known that the wall of Gram-positive bacteria simply consists of several layers of peptidoglycan, while the wall of Gram-negative bacteria has an additional hydrophilic outer wall; for this reason EOs components, which are mainly hydrophobic, find it difficult to penetrate inside the Gram-negative cells [61]. Thus, authors demonstrated that the presence of hydrophobic terpenes is critical for Gram-positive antibacterial action, as previously stated by Cox [62]. Moreover, terpenes’ mechanism of action was verified in altering the permeability of the membrane by interacting with the lipid components, thus inducing cell death [62]. A similar mechanism of action was found for *Cinnamomum glanduliferum* EO [63], tested against some Gram-positive and Gram-negative bacterial strains, and also some fungi. Through the transmission electron microscopy (TEM) assay performed by Taha et al. on *E. coli*, there emerged an irreversible damage in the cell membrane. According to the evidence obtained from this study, another plausible mechanism of action involves the blockage of the production of some important enzymes, such as protease and amylase, leading to cell components coagulation [64,65,66,67]. These phenomena are also used by fungi, which are able to cause the destruction of the cell membrane even through the ability to inhibit the production of cellular energy and to create a pH gradient between the cell and the external medium. These phenomena, in this particular EO context, are caused by the great amount of eucalyptol, and also due to the significant presence of other terpenes (terpinen-4-ol and α-terpineol); these three major compounds, as the author suggested, were responsible for the antimicrobial activity of *Cinnamomum glanduliferum* EO. Insawang and co-workers compared five cultivars of *Lavandula stoechas*, in order to assess the differences in their antimicrobial behavior against some bacterial strains [68]. This study is a further confirmation that the presence of eucalyptol (and terpenes in general) is crucial for the antimicrobial activity of the EO. Thymol and carvacrol have been investigated for their antimicrobial properties by Pesavento and coworkers [69], who reported the antimicrobial activity of *Origanum vulgare* EO on opportunistic respiratory pathogens. In this work, 59 bacterial strains belonging to *Staphylococcus aureus*, *Stenotrophomonas maltophilia*, and *Achromobacter xylosoxidans* species were subjected to the treatment with *Origanum vulgare* EO [69]. All strains resulted to be highly susceptible, even in the treatment with low EO concentrations. In particular, a 48-h-long treatment with a 0.5% *v/v* concentration of the EO was able to completely inhibit (100%) the growth of *A. xylosoxidans*, *S. maltophilia* and multi-resistant *Staphylococcus aureus* (MRSA). Interestingly, the selected strains belonged to different bacterial species or genera, and it emerged that none of them muted becoming resistant: these findings suggested that the broad activity of *Origanum vulgare* EO could be achieved through the interaction with multiple cellular targets, thus minimizing the possible occurrence of mutant-resistant strains. These results encourage a possible use of *Origanum vulgare* EO in the treatment of multidrug-resistant cystic fibrosis (CF) pathogens, as suggested by the authors [69].

*Thymus sipyleus* was investigated to demonstrate its effectiveness in the treatment of rhinosinusitis, as already used in traditional medicine [70]. Eight bacterial strains—*Staphylococcus aureus*, methicillin-resistant *S. aureus* (MRSA), *S. epidermidis*, *S. pyogenes*, *S. pneumoniae*, *P. aeruginosa*, *M. catarrhalis*, and *H. influenzae*—were analyzed using three different methods. Comparing with ampicillin, clarithromycin, chloramphenicol, and amoxicillin/clavulanic acid (1:1) as positive controls, the agar diffusion method demonstrated that all bacterial strains were susceptible, with a maximum value for *S. aureus*; the only exception was *P. aeruginosa*, however this result is related to the higher resistance of Gram-negative bacteria compared to Gram-positive ones, as previously described [3,65,71,72,73]. The microdilution method has shown that the EO showed an excellent antimicrobial activity on these species at all the tested concentrations, though the effectiveness was lower than one of the positive controls. The vapor diffusion method has also produced encouraging results, especially by demonstrating good action against MRSA strain. The antimicrobial activity of EOs can be exploited in the treatment of skin injury infections. In fact, the presence of the wound causes the reduction in the primary barrier effect, performed by the skin toward the external environment and, as the most dangerous consequence, an increased risk of exposure to microbial infections [65,74,75,76]. EOs have been recognized as a valid therapy in injured skin infections for their proven antimicrobial activity against multidrug-resistant skin pathogens; however, at certain concentrations, EOs could be cytotoxic, so the risk-benefit ratio for each one should be previously assessed [77]. Another feature supporting the topical use of EOs is their ability to promote wound healing. This potential was discovered by the French chemist Rene-Maurice Gattefossé about a century ago: an explosion in his laboratory caused a severe burn to his hand, but the immersion in lavender EO provided a quick healing, without infection or scar marks. Since that period, many studies aimed at deepening the biochemical basis of this phenomenon, in order to confirm a possible use of EOs for skin healing. Wound healing is composed by three phases: The first one is the inflammatory phase, which includes bleeding blockage, then vasodilation and immune system recruitment [78]; the second phase involves the proliferation of various cell lines, including fibroblast, leading to tissue granulation and angiogenesis [79,80,81]; finally, in the third phase new collagen fibers are generated and fibroblasts differentiate, bringing the two edges of the wound closer to each other [78,79,80,81,82]. Costa and co-workers demonstrated that the presence of carvacrol and thymol, the most widespread monoterpenes, is relevant in determining EO tissue repairing activity [75]. In fact, among their various biological activities, they can modulate all phases of tissue regeneration, especially the first one, because of their great anti-inflammatory action. Moreover, due to their remarkable anti-inflammatory action, sesquiterpenes are also able to promote tissue repair, eventually in synergy with monoterpenes [83]. Recently, Seyed Ahmadi et al. illustrated the biochemical mechanisms that permit wound healing through the topical administration of *Cinnamon verum* EO, using a mice model of wound infected by *Staphylococcus aureus* and *Pseudomonas aeruginosa* [84]. Besides the proven antimicrobial activity of the EO, also its antioxidant and anti-inflammatory activities were found relevant, because the shorter the inflammatory phase lasts, the faster the proliferative process that leads to wound healing begins. Furthermore, the three main biochemical mediators modulated by the EO were identified: IGF1, whose expression was enhanced, leading, among other implications, to an increase of fibroblast cells and proliferation; VEGF and FGF-2, whose biosynthesis and expression were up-regulated, respectively, improving the angiogenesis, which is important in various stages of inflammation and cell proliferation. *Melaleuca alternifolia* was investigated by Edmondson and co-workers to assess its antimicrobial and wound-healing activity [85]. The volunteers, who had wounds infected with Methicillin-resistant Staphylococcus aureus (MRSA), were medicated for a few days with an EO solution: the antibacterial action of the EO toward this resistant bacterial strain was demonstrated. Moreover, the reduction in size of the wound was found significant according to the analytical method used, thus authors demonstrated that the EO is able to promote the wound healing. Wounds infected with MRSA were also subjected to the treatment with *Anethum graveolens* EO, obtaining many important results [86]. Antimicrobial activity was demonstrated by the significant reduction in bacterial growth. In addition, this EO proved to be able to influence various phases of wound healing. In the inflammatory phase, it increased the expression of p53 and caspase-3, which respectively promote apoptosis and stimulate the proliferation of stem cells to repair tissue [87]. Moreover, this EO up-regulated the expression of Bcl2, an anti-apoptotic agent, as well as VEGF and FGF-2, which have important roles in angiogenesis and epithelial cells proliferation and differentiation [88,89,90]. Finally, it influenced the expression of Erα-promoting collagen biosynthesis. These results demonstrated the potential use of *Anethum graveolens* EO in the treatment of infected wounds, due to the healing properties related to the presence of its major compounds, α-phellandrene, p-cymene, and carvone. *Lavandula angustifolia* EO has also proven to have great beneficial effects on wounds. Mori and co-workers demonstrated that the topical application of this EO on wounded mice caused a faster closure of the lesion compared to the untreated and control groups, suggesting its potential use in the initial stages of tissue regeneration [91]. Moreover, the ELISA analysis showed a significant augmentation of TGF-β levels, which promotes wound contraction through increasing the number of fibroblasts, thus resulting in an intensified collagen production. Finally, it emerges that *Lavandula angustifolia* EO caused a quicker switch from collagen type III to collagen type I. All the cited studies therefore showed that EOs for topical use may constitute, in the close future, the alternative therapy for the treatment of chronic and infected wounds.

### 3.3. Anxiolytic Activity

Since past ages, it is well-known that natural substances are able to affect the psychic functions of an individual [92]. In literature an impressive number of information is reported about the plants employed to treat symptoms related to the most common psychiatric disorders [93,94]. Among these, it is noteworthy to mention the mandrake, whose magical qualities were showed during the Middle ages, probably for its sedative-hypnotics effects or the hellebore, a drug known to treat paranoia, epilepsy [92,95], and also hysterical suffocation which today refers to “functional neurologic symptom disorder” [96]. In this context, it is remarkable to mention the important traditional drugs extracted from plants, able to affect the central nervous system (CNS). For instance, reserpine isolated from *Rauwolfia serpentina*, has revolutionized schizophrenia treatment [97], as well as morphine, derived from *Papaver somniferum*, which acts to decrease the feeling of pain [98]. In the past few years, the competition between synthetic and natural drugs has led to the re-evaluation of the plant world, with a significant increase in pharmaceutical plantation investments. In this recent scenario, EOs represent a new challenge by means of their pharmaceutical applications to improve physical, mental, and emotional well-being. The beneficial effects of EOs are achieved by administering (e.g., inhalation, ingestion or cutaneous application) these substances within therapeutic doses, but if these dosages are exceeded, the effects may be harmful to the human body [99]. Inhalation is most commonly used in the acute administration [100]. Remarkably, olfactory system is considered the primordial sensory system in animals: its role is the identification of mixed molecules, present in the environment, to capture new information. To date, the most supported mechanism about olfactory system was described by Linda Buck and Richard Axel. Accordingly, they showed that the detection of distinct odorants may be the result of the association of odorous ligands with specific receptors on olfactory sensory neurons [101]. In particular, through the inhalation, odorant molecules combine with olfactory receptors (ORs) located on the nasal olfactory epithelium, whence the transmission of signals start from olfactory tracts to the olfactory bulb which, in turn, has a link to the limbic system- mostly with amygdala, responsible for emotional regulation, and hippocampus, involved in memory processes [35]. Therefore, emotional effects of smells can include fear, anxiety, aversion, as well as pleasure or relaxation, through releasing neurotransmitters, such as endorphins, producing a sense of well-being [102]. For this reason, recent scientific reports suggested benefits in aromatherapy. Accordingly, it is not surprising that today a PubMed search with the keywords “essential oil” results in more than 22,700 publications. In this regard, it is noteworthy to mention Marguerite Maury, one of the first researchers demonstrating the potential effects of EO on the nervous system, mostly in the limbic system [99]. Nowadays aromatherapy is practiced in several countries, including UK, France, USA and Australia, as “*complementary therapy*” in association with the traditional medicine. The desire to re-evaluate some traditional forms of medicine as integrative remedy has recently been expressed by the World Health Organization (WHO) (World Health Organization. WHO Traditional Medicine Strategy: 2014–2023, Geneva: WHO; 2014). Considering the effects of EOs at the CNS and according to a plethora of evidences [103,104], the use of EO as supplementary treatment can be a powerful coadjuvant in the care of patients with anxiety disorders [105]. Anxiety disorders, in its pathological meaning, represent one of the most common classes of psychiatric disorders, which impact negatively the quality of people’s life [106]. According to the Diagnostic and Statistical Manual of Mental Health Disorders, anxiety disorders can be classified into several main types: generalized anxiety disorder (GAD), panic disorder, specific phobia (SP), agoraphobia, selective mutism, social anxiety disorder (SAD) or social phobia, separation anxiety disorder, as described by the American Psychiatric Association. Common comorbidities include mood substance and personality disorders [107]; moreover anxiety disorder affects women more easily than men [100]. Psychological, environmental, biological, and genetic factors represent the main causes of predisposition to anxiety disorders [108]. Currently the first and second line treatments for anxiety include *benzodiazepines* (BZD), *selective serotonin reuptake inhibitors* (SSRI), *serotonin norepinephrine reuptake inhibitors* (SNRI), *tricyclic antidepressant* (TCA), *monoamine oxidase inhibitor* (MAOI), up to the most recent atypical antidepressants. Furthermore, innovative compounds are being developed for glutamate, neuropeptide, and endocannabinoid systems [109]. Although many efforts have been made, unfortunately the most anxiolytic drugs, even if effective, can cause a series of side effects, as well as induction of a state of dependence and tolerability problems. Moreover, another obstacle in the development process of anxiety drugs includes lack of the full understanding of anxiety disorders related physiopathology [110]. For these reasons, it is essential to consider the additional effective treatments. The use of EOs would seem a promising way to reduce mild anxiety symptoms or at least to potentiate the effect of traditional anxiolytic approaches: accordingly, an innovative “pharmaco-ethology” approach attempting to find drugs with new mechanism of action is needed [111]. Most of the EOs have been proven to be anxiolytic in clinical trials as well as in animals through the most reliable behavioral models, such as open field (OF) test [112], elevated plus maze (EPM) test [113], social interaction (SI) test [114], light and dark box (LDB) test [115], marble-burying (MB) test [116], etc., and pharmacological approaches to induce anxiety in mice or rats [117]. Among all the studies reported, *Lavandula angustifolia* (the most used species of lavender) is widely studied also for its anxiolytic effects [118]. The exposure to lavender smell showed an anxiolytic profile similar to diazepam, mostly in female gerbils [119]. Investigation of the effects of inhaled linalool, a monoterpene commonly found in several aromatic plant, as well as in *Lavandula angustifolia*, showed anxiolytic properties in mice, increased social interaction, and decreased aggressive behavior [120]. Results showed that the inhalation of *L. angustifolia* EO had an anxiolytic effect in rats with decrease of freezing, as well as a reduced expression of c-Fos in prefrontal cortex and amygdala [121]. Shaw et al. compared the effects of lavender oil with the chlordiazepoxide (CDP), as anxiolytic reference, suggesting that lavender oil has anxiolytic effects on rats during the open field test, but that a sedative effect can also occur at the highest doses [122]. Interestingly, there are also some clinical evidences, which confirm in vivo studies. Karan et al. (Germany), described that the anxiolytic effect of lavender oil inhalation was able to reduce peri-operative anxiety in patients undergoing surgical procedures under local anesthesia [123]. Kritsidima and co-workers, in a randomized controlled trial on patients, described how the lavender scent reduces state anxiety in dental patients [124]. A pilot study on the effects of lavender oil on anxiety and depression in the high risk postpartum woman showed a significant improvement of the Edinburgh Postnatal Depression Scale and Generalized Anxiety Disorder Scale after four consecutive weeks of lavender administration [125]. In another study on forty-five adult outpatients which met the DSM-5 criteria for major depression, anticholinergic side effects of imipramine, such as dry mouth and urinary retention, were observed less often when lavender was administered with that drug, suggesting an effective adjuvant therapy in combination with this latter [126]. A study including 40 students with Premenstrual Syndrome (PMS) problems and 37 students as control groups showed that lavender inhalation could be used to reduce symptoms of PMS, such as anxiety [127]. Several studies reported pharmacological properties of lavender EO to interact with neuropharmacological targets. Chioca et al. described that serotoninergic transmission, in particular 5-HT1_A_ receptors, could play an important role in the anxiolytic-like effect of lavender EO. Surprisingly in the same study lavender oil inhalation appeared to attenuate the serotoninergic syndrome induced by SNRI [128]. However, other findings suggested that the serotonergic transmission via 5-HT1_A_R may not be involved in the anxiolytic effects induced by linalool odor: a potential role of GABAergic transmission was described, especially on GABA-A receptors, enhancing the inhibitory tone of the nervous system [129]. Other papers suggested a novel mechanism, compared with traditional anxiolytic drugs, in which linalool and linalyl acetate displayed inhibitory activity on Ca^2+^ influx mediated by voltage-dependent *calcium channels* (VGCCs) [104]. To date in Germany, the EO extract of *L. angustifolia* for oral administration has been developed, approved and it is a registered drug for the treatment of restlessness accompanying anxious mood. The SLO product (Silexan, W. Spitzner Arzneimittelfabrik GmbH, Ettlingen, Germany) contains the two primary constituents of lavender oil—linalool and linalyl acetate—at concentrations of 36.8% and 34.2%, respectively [105,130,131]. This drug has significant anxiolytic and sleep-improving effects not associated with sedation [132] and also the absence of potential dependency [133]. Lavender, as well as a lot of EOs (Table 2), is “generally recognized as safe” (GRAS) as a food by the U.S. Food and Drug Administration. In general, lavender is well tolerated, but more information is needed, as well the safety on the excretion of some lavender components into breastmilk. Because different or also identical chemotypes can have very different chemical components, it is not surprising that linalool is not the only one having anxiolytic action (Table 2). According to the available clinical studies, *Citrus aurantium* EO on the treatment of anxiety, in patients with chronic myeloid leukemia (CML) [134], as well as in patients with acute coronary syndrome (ACS) [135] showed a reduction of symptoms associated with anxiety. Also, *Citrus sinensis* and *Citrus bergamia* EO demonstrated positive effects against signs of mild anxiety [136]. In another studies the researchers assessed the effect of *Rosa damascene* EO, showing a reduction of anxiety and pain in the first stage of labor [137], as well as *Osmanthus fragrans* oil and grapefruit oil were effective complementary treatments for anxious patients undergoing colonoscopy [138]. Tankam and co-workers investigated the potential of *Piper guineense* EO in mice, demonstrating that its inhalation could induce a mild tranquilizing effect [139]. Other studies suggested that lemon oil possesses anxiolytic and antidepressant-like effects, probably through the suppression of DA activity related to enhanced 5-HTergic neurons [140]. Zhang et al. showed an anxiolytic effect on male mice after inhalation of *Cananga odorata* EO (ylang-ylang EO) [141]. Another study analyzed the potential anxiolytic properties of inhaled coriander volatile oil, extracted from *Coriandrum sativum*, on a rat model of Alzheimer’s disease [142]. Forms of emotional memory associated to anxiety states were observed in a pilot study on postpartum women, demonstrating a reduction of anxiety levels using a rose/lavender oil blend for 15 min twice weekly during 4 weeks [125]. Even if our purpose is to discuss about aromatherapy, it is noteworthy to admit that all the anxiolytic-like effects in animal models have been obtained also through oral or IP administration [143,144,145,146]. Evidence suggests that aromatherapy might be used as a complementary treatment, however some papers seem to support the ineffectiveness of Eos. For instance, Fazlollahpour and co-workers described that inhalation of aromatherapy with 4% rose EO could not significantly alleviate anxiety in patients undergoing coronary artery bypass graft (CABG) surgery [147]. Another study trials conducted in 2007 did not demonstrate a statistically significant relationship between the use of EOs and anxiety reduction [148].

## 4. EOs Encapsulation Strategies in Drug Delivery Systems

**Figure 2:**
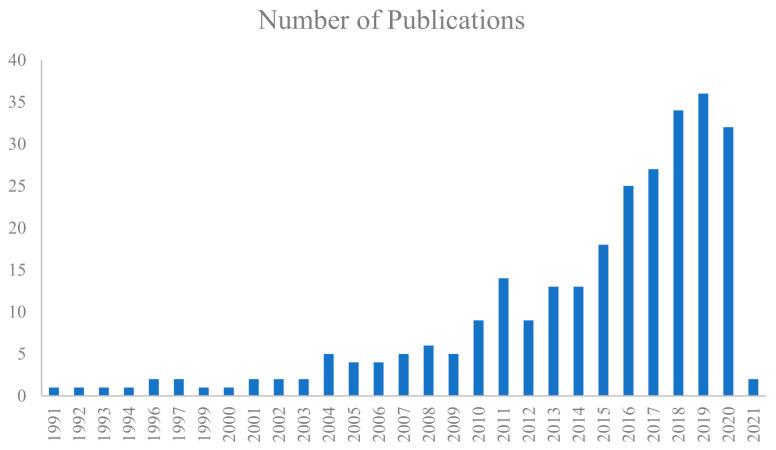
Number of published research articles searching the keywords “essential oils” and “delivery” and “pharmaceutical”, source PubMed, last update 12 January 2021.

Despite many interesting applications, the EOs are fragile and unstable volatile compounds, subject to enzymatic reactions, phenomena that compromise their biological properties, causing a decrease of activity and an increase of toxicity (irritation, photosensitization, etc.,), which limit their traditional use. These limits could be improved exploiting the encapsulation of EOs in drug delivery systems (DDS) that provide a controlled drug release, increasing the bioavailability and efficiency of EO, thus providing an optimal pharmacokinetic profile [6]. Literature production on the encapsulation of EOs mostly deals with food, textile, and cosmetic industry [149], even if in the recent decade the interest in the pharmaceutical field has raised, as demonstrated by the increasing number of published papers concerning the delivery of EOs for pharmaceutical applications (Figure 2).

**Figure 3:**
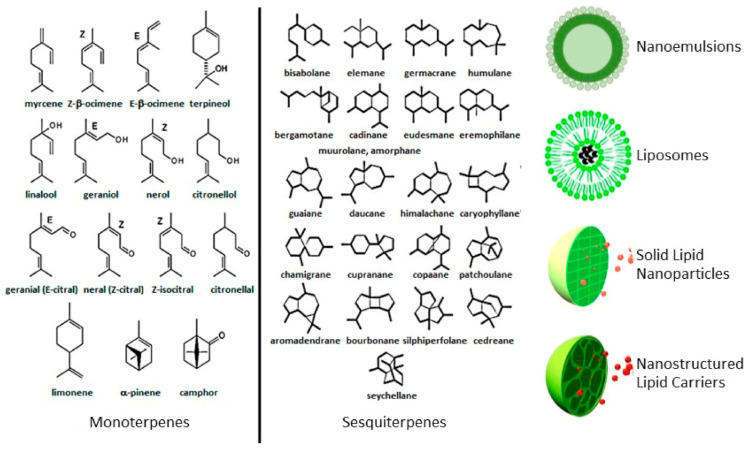
Main lipid-based delivery systems for EOs delivery. Reproduced with permission from [151].

Many advantages can be listed when exploiting EOs encapsulation in DDS, such as: increased bioavailability (due to the ability of DDS to adhere to the mucous membrane); protection from hydrolysis and oxidation, thus increasing EO chemical stability; reduction of toxicity and volatility; the possibility of reaching target sites with therapeutic doses, thus increasing the patient’s compliance. Different strategies related to the use of DDS have been explored for the potential encapsulation of EOs, including polymeric nanoparticles and inclusion complexes with cyclodextrins, which have been extensively reviewed [6,150]. Herein, we focus on vesicular and nanoparticulate lipid-based delivery formulations, such as micro- and nanoemulsion, liposomes, solid lipid nanoparticles (SLN), and nanostructured lipid carriers (NLC) (Figure 3).

### 4.1. Micro- and Nanoemulsions

Microemulsions (mean droplet size 100–400 nm [152]) and nanoemulsions (1 to 100 nm [153]) are isotropic dispersions of two non-miscible liquids (oil and water). The former is thermodynamically stable system in which the inner phase can figure as spheroid, cylinder-like, plane-like, or as a bicontinuous structure [154], whereas the latter is thermodynamically unstable, usually requiring a co-surfactant because its free energy is higher than the free energy of the separate oil and water [155].

**Figure 4:**
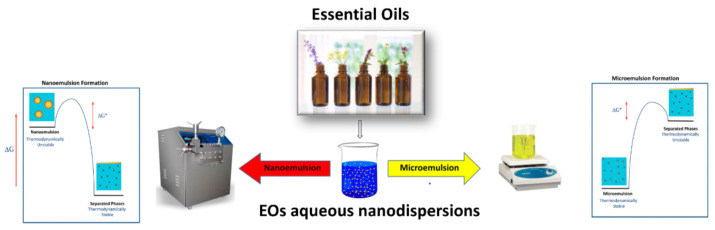
Nanoemulsions and microemulsions s for EOs delivery. Reproduced with permission from [157].

A range of different EOs formulated both in micro- and nanoemulsions have demonstrated certain antimicrobial, antiviral, and antifungal activities [156] (Figure 4).

EOs have also demonstrated analgesic, anti-inflammatory, antioxidant, antibacterial, anti-fungal, sedative, and antidepressant effects, and through topical application, can effectively heal burns and insect bites [158], therefore they can be used in the treatment of skin wounds. This effect is related to their ability to damage the bacterial cell wall or cell membrane, thus increasing cell membrane permeability and solubilization, with the consequent release of membrane proteins, leading to bacteriostasis [159]. Despite the very promising medicinal activities, some factors limit the pharmaceutical application of EOs as antimicrobial agent in topical treatment, mainly because their directly exposure to the skin could potentially induce an allergic reaction. Moreover, EOs are highly instable and easily degrade when exposed to external factors (oxidation, evaporation, heat, and light) [6]. Furthermore, low aqueous solubility and high volatility limit their free use without a pharmaceutical vehicle. One of the ways to overcome these limits is the use of encapsulation strategies, into vesicular or particulate delivery systems. One strategy consists in the use of micro or nanoemulsions. These vesicular nanosystems have been demonstrated to increase the EOs bioavailability and diffusion—thanks to their small droplets size—and contribute to the antimicrobial and anti-biofilm activities—due to the wetting ability of surfactants [160], since the high surface tension of the droplets favors their fusion with the membranes of prokaryotes, viruses and eukaryotic cell of fungi, leading to the destruction of these organisms [161]. Interestingly, Donsì et al. demonstrated that the nanoencapsulation of terpenes extracted from *Melaleuca alternifolia* improved the antimicrobial activity compared to the unencapsulated mixture, but they also reported that the theoretical higher delivery efficiency of nanoemulsion loaded with terpenes, associated with its smaller mean droplet diameter, is likely counter-balanced by the partial degradation of some active compounds, and in particular, carvacrol [162]. Sugumar and co-workers studied the antimicrobial effect of *Eucalyptus* EO, both in pure form and encapsulated into nanoemulsions [163]. The antibacterial studies of nanoemulsion obtained by sonication against *Staphylococcus aureus* by time kill analysis showed complete loss of viability within 15 min of interaction, thus confirming a high interaction with the bacterial membrane and therefore an increase in antimicrobial activity. The integrity of the bacterial membrane was analyzed in the range 0–60 min, showing an immediate loss of cytoplasmic contents of 83.32%. Interestingly, wound contraction activity studies showed better wound-healing activity in animals treated with nanoemulsion (100% wound healing on day 16), compared with the commercially available drug neomycin treated (94.2% healing rate) and control rats, probably due to the ability of the droplets to form a film on the skin after the evaporation of nanoemulsion water phase, and to the presence of cineole in the eucalyptus oil, a well-known penetration enhancer in transdermal DDS and topical application studies [164]. Alam and co-workers investigated the wound contraction activity of clove EO in nanoemulsion by in vivo studies on rats, in comparison to clove EO pure treatment and positive control treated with gentamycin [165]. The collected results demonstrated that the wound contraction and epithelialization time obtained with clove EO-loaded nanoemulsion and gentamycin were comparable, while EO-loaded nanoemulsion results to be more effective than the pure clove EO. The authors also investigated the wound contraction activity of nanoemulsions loaded with *Eucalyptus* EO [166]. Again, the effectiveness of the EO-nanoemulsion was statistically higher compared to the pure EO, reaching epithelization time results similar to those obtained in the treatment with gentamycin. All the results obtained by Alam and co-workers underline that EO encapsulation into nanoemulsion allows increasing the effectiveness in the wound-healing treatment. Recently, the potential application of *Lavandula angustifolia* EO and licorice extracted from *Glycyrrhiza glabra*, in a nanoemulsion delivery system was investigated [167]. In this work, Kazemi et al. demonstrated that this formulation strongly improved the wound-healing process at different stages (wound closure, epithelialization, and molecular processes). In particular, nanoemulsion loaded with *Lavandula angustifolia* EO and licorice extract was able to increase the expression of TGF-β1, type I and type III collagen genes, which are involved in the acceleration of the wound-healing process. The authors attributed this effect to the role of *Lavandula angustifolia* EO on the stimulation of TGF-β1, an important regulatory factor in reproduction fibroblasts which is involved in the formation of granular tissue as well as in the synthesis and accumulation of collagen fibers [167]. EOs-loaded nanoemulsions have also been studied for their potential anti-inflammatory activity by in vitro and in vivo (in zebrafish) assays [39,168]. In particular, Borges et al. focused on the anti-inflammatory activity of nanoemulsions loaded with *Rosmarinus officinalis* L. EO, exploiting two parameters: the cellular antioxidant activity (CAA) and the nitric oxide (NO∙) production. Interestingly, authors found that the values obtained from the antioxidant activity studies of the pure EO were not significantly different to the one of the respective nanoemulsion, even if the concentration of *Rosmarinus officinalis* L. EO in the nanoemulsion was three times lower compared to the pure EO. The obtained results were correlated to the lipophilicity of the nanoemulsion droplets, which facilitate the penetration into the cell membranes thus promoting the release of EO. Furthermore, Borges et al. demonstrated that *Rosmarinus officinalis* L. EO was able to induce a dose-dependent reduction of NO∙production, whereas a dose-independent effect was observed for nanoemulsions whose activity was greater than pure EO [39]. Therefore, authors concluded that the produced nanoemulsions was able to enhance the anti-inflammatory effect of EO, further reducing the production of NO∙. In particular, the potential pro- or anti-inflammatory effect of EO was investigated on human keratinocytes and fibroblasts, in order to evaluate their safe use for operators [168]. Herein, Rossi et al., as well as proving that EOs present a strong toxicity against larvae and pupae of the main malaria vectors *An. stephensi* and *An. gambiae*, in Asia and Africa, also demonstrated that the CBD-free hemp EOs did not induce an inflammatory condition. In particular, these compounds were able to revert the inflammation, reducing the release of the cytokine on skin cell lines, thus demonstrating that they could be safely used by operators [168].

### 4.2. Liposomes

**Figure 5:**
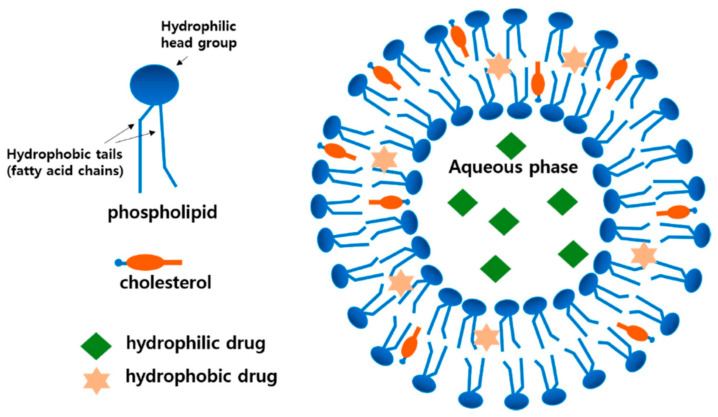
Liposome’s structure characterized by a spherical vesicle with a phospholipid bilayer membrane used to deliver hydrophilic or hydrophobic drug. Reproduced with permission from [170].

Liposomes are vesicular self-assembled system characterized by one or more bilayers, constituted by phospholipids, surrounding an aqueous core. They were first studied as drug delivery systems in 1970, since they can be used as carriers for both lipophilic and hydrophilic molecules, due to the presence of the hydrophilic compartment and lipophilic bilayer [169] (Figure 5). The encapsulation of an active compound into liposomes allows both the protection from degradation and the increase of solubility.

Different studies demonstrated the potentiality of liposomes in the delivery of EOs. Valenti et al. reported that *Santolina insularis* EO showed a good in vitro antiherpetic activity against Herpes simplex virus type 1 (HSV-1) and that its stability was improved when loaded into liposomes [171]. The same research group also developed multilamellar vesicles (MLV) and small unilamellar vesicles (SUV) to improve the antiviral properties of *Artemisia arborescens* L. EO [172]. Interestingly, authors found that EO-loaded MLVs enhanced the antiviral activity against HSV-1, while no significant differences of the antiviral activity were observed between the free EO and SUV vesicles, which showed a poor activity as determined by both the reduction of viral cytopathic effect assay and plaque reduction assay. Therefore, they concluded that the incorporation of *A. arborescens* EO in multilamellar liposomes greatly improved its activity against intracellular HSV-1 [172]. Liolios and co-workers further investigated the influence of the liposomial encapsulation on the antimicrobial activity of *Origanum dictamnus* L. EO [173]. Carvacrol, thymol, p-cymene, and c-terpinene, identified as the major constituents of *Origanum dictamnus* L., were isolated and successfully encapsulated in phosphatidyl choline-based liposomes, in order to evaluate the potential improvement of their antioxidant and antimicrobial activities against four Gram-positive and four Gram-negative bacteria and three human pathogenic fungi, as well as the food-borne pathogen, *Listeria monocytogenes*. Interestingly, authors found that the encapsulation of monoterpenes leads to vesicles’ stabilization, as demonstrated by the increase in the onset temperature. Furthermore, a significant increase of the antimicrobial activity was found after the encapsulation in liposomes [173]. Liposomal formulation loaded with *Zataria multiflora* Boiss EO, a well-known aromatic medicinal herb in Persian as “Avishan Shirazi” or “Azkand”, were successfully produced in order to compare the antibacterial activity of free EO and EO-loaded liposomes against *E. coli* [174]. Authors found that MIC and MBC values of EO-loaded liposomes were 1.4% and 1.8%, respectively, thus demonstrating the increasing antibacterial activity of the EO when loaded into liposomes. This result was related to the ability of liposomal formulation to improve the cellular transport and to release the active components inside the cell, due to cells interaction (inter-membrane transfer, contact release, absorption, fusion, and phagocytosis), whose mechanism is related to the cell type (cell wall/membrane composition), as well as liposome membrane’s physicochemical properties, as previously reported [175]. Sebaaly et al. investigated the encapsulation of clove EO in natural soybean phospholipid liposomal vesicles, prepared by using the ethanol injection method [176]. Authors demonstrated that liposomes were able to protect eugenol, the main component of clove EO, from the degradation induced by UV exposure, without any reduction of its DPPH scavenging activity. Interestingly, the same authors also investigated the possibility to prepare clove EO and eugenol-loaded liposomes using large scale procedures, such as membrane contactor [177,178]. The potential antibacterial activity against *E. coli* was demonstrated by Najafi et al. for liposomes prepared with lecithin/cholesterol and loaded with barije EO, extracted from *Ferula gummosa* [179]. An interesting application of EO-loaded liposomes was recently investigated by Palmas et al. [180]. Herein, liposomes prepared with *Citrus limon* var. *pompia* were prepared as potential mouthwash product for the treatment of oropharyngeal diseases. Citral-loaded liposomes were demonstrated to be biocompatible and able to protect cells from the damages caused by oxidative stress. The vesicles also demonstrated to promote the healing of wounded mucosa, favoring the closure of lesions induced in a keratinocytes cell monolayer, and to inhibit the proliferation of *Streptococcus mutans*, when loading 50 mg/mL of citral EO [180]. An interesting approach was recently developed basing on drug-in-cyclodextrin-in-liposomes (DCLs), which are constituted by one or more phospholipid bilayers and an aqueous internal cavity where a cyclodextrin/drug inclusion complex is loaded [181]. Herein, Hammoud et al. encapsulated different EO components (estragole, eucalyptol, isoeugenol, pulegone, terpineol, and thymol) into DCLs, prepared by the ethanol injection method using lipoid S100 and hydroxypropil-β-cyclodextrin, as a promising tool for extending EOs shelf life and activity. The physico-chemical and technological characterization of DCLs revealed their ability in significantly improving the loading ratio of estragole, pulegone, and thymol, and prolong the release of EO components compared to traditional liposomes [181,182]. Recently, Lin and co-workers developed an interesting strategy for the encapsulation of thyme EO into solid liposomes coated with ε-polylysine [183]. Solid liposomes are novel nanosystems, with higher stability and longer storage time compared with traditional aqueous liposomes [184].

### 4.3. Lipid Nanoparticles: SLN and NLC

**Figure 6:**
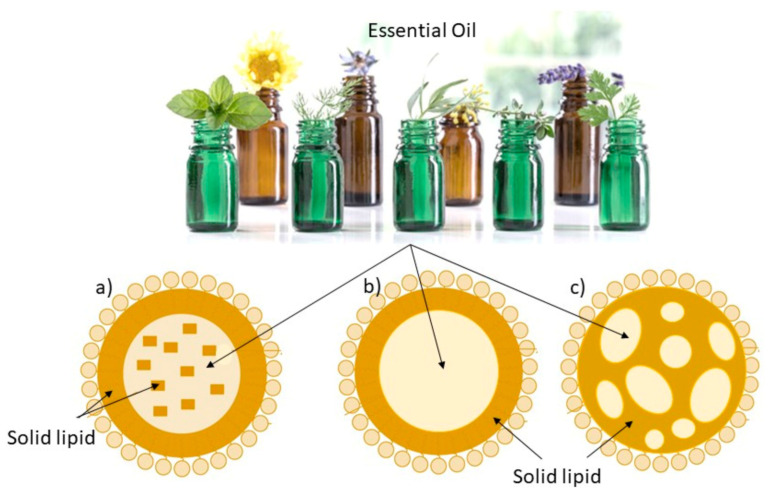
EOs delivery into solid lipid nanoparticles (SLN) (**a**) or nanostructured lipid carriers (NLC) of different types, depending on the formation of a single (**b**) or different nanocompartments of oil (**c**).

SLN were proposed in the early 1990s as a tool to overcome the limits related to liposomes, exploiting the use of physiological lipids (e.g., blends of mono-, di- or tri-glycerides, fatty acids, waxes) [185], solid at room and/or body temperature, in mixture with surfactants and water. The solidity of the lipid attributes some advantages to the system, such as increased chemical protection, less leakage, and sustained release [186]. Other relevant advantages are the high targeting effect and low toxicity, which is related to the use of biodegradable and biocompatible lipid substances with a GRAS (“generally recognized as safe”) status [187]. Different studies on EOs-loaded SLN report the enhanced anticancer activity of EOs [188]. Frankincense and myrrh oil obtained from the genera *Boswellia* and *Commiphora*, respectively, were loaded into SLN prepared with Compritol 888 ATO by high-pressure homogenization, and their anticancer activity after oral administration was evaluated in vivo in H22-bearing Kunming mice [189]. The obtained results demonstrated the possibility to encapsulate EO into SLN having mean size smaller than 220 nm, with high encapsulation efficiency (>98%). Furthermore, a significantly higher in vivo antitumor efficacy was observed compared to EO suspension and EO-loaded into β-cyclodextrin, used to increase EO solubility, at the same dosage. Another interesting anticancer application was recently proposed by Rodenak-Kladniew and co-workers based on the delivery of linalool into SLN prepared with different solid lipids (myristyl myristate, cetyl esters and cetyl palmitate) prepared by sonication using Pluronic^®^F68 as surfactant [190]. The aim of this work was to exploit the potential anticancer activity of linalool (an acyclic monoterpene alcohol commonly found in EOs of different plants and herbs including lavender, basil, rosemary, citric fruits, green, and black tea) as a mono-drug agent or in combination with traditional drugs. Collected results demonstrated the possibility to obtain stable formulations with mean size around 100 nm, able to provide a controlled release of linalool EO. Interestingly, a cytotoxic activity was observed against human lung- and liver-derived tumor cells (A549 and HepG2, respectively), thus demonstrating that linalool-SLN represents a potential combined approach to enhance the activity of anticancer drugs [190]. Another application of EO-SLN was investigated by Zhao et al., who focused their research on the pulmonary delivery of *Yuxingcao* EO in SLN prepared with Compritol 888 ATO by high-shear homogenization, using polyvinyl alcohol as emulsifier agent, thus overcoming the rapid clearance of pulmonary absorption [191]. Authors demonstrated the feasibility of the encapsulation strategy to produce a *Yuxingcao* EO-SLN inhalation delivery able to improve the elimination half-life in the lungs, enhancing the active compound local bioavailability, thus potentially limiting the administration doses to one per day. Due to the antimicrobial activity of EOs, their encapsulation into SLN has also been investigated as a promising strategy to reduce the microbial resistance to antibiotics, one of the major problems in the treatment of different diseases, such as topical infections, and in wound healing. In order to improve the antibacterial and antifungal activity of clove oil (the EO obtained from *Eugenia caryophyllata*), Fazly et al. prepared SLN by high-shear homogenization and ultrasound method, using glyceryl monostearate, precirol, and stearic acid as lipid phase [192]. Clove encapsulation into SLN effectively improved its antimicrobial activity, reducing the MIC/MCC values from 2–20 folds, with higher effects against Gram-negative bacteria and fungi. This result was related to the ability of SLN to interact with microbial cell membrane and to the improvement of the EO stability and solubility [192]. Authors observed that clove-loaded SLN prepared with stearic acid showed better results compared to SLN prepared with the other selected lipids, and related this result to its lower particles size which would increase passive cellular absorption enhancing antimicrobial activity, as reported by Nasseri et al. [193]. SLN can be also used as carrier for more than one antimicrobial drug: in order to enhance the antimicrobial activity of ofloxacin against *Pseudomonas aeruginosa* and *Staphylococcus aureus*, Rodenak-Kladniew prepared a SLN platform containing the aforementioned drug together with two antibacterial active components, chitosan and eugenol, considering various matches: ofloxacin, ofloxacin-SLN, ofloxacin-chitosan-SLN, ofloxacin-chitosan-eugenol SLN. Considering the *Pseudomonas aeruginosa* model, the obtained MIC values went from about 1.73 ± 0.40 μg/mL of free ofloxacin to 0.29 ± 0.07 μg/mL of ofloxacin-chitosan-eugenol SLN, thus causing a six-fold decrease of this parameter. Even better results emerged for *Staphylococcus aureus* model, with a 16-fold reduction of MIC values, from 0.31 ± 0.05 μg/mL of free ofloxacin to 0.02 ± 0.01 μg/mL of ofloxacin-chitosan-eugenol SLN. This excellent microbial toxicity is not associated with toxicity to human cells, as shown by the MTT assay. On the other hand, with concentrations 10–150-fold higher than the ones required for therapeutic action, eugenol alters glutathione levels leading to cell death [190]. The effect of physicochemical properties on the different activities of nanoparticulate systems was also investigated by Pereira et al. [194], who developed and optimized, using factorial design, linalool-SLN with long-term stability, demonstrating that the variation of surfactant concentration significantly influences mean particles size and homogeneity, since as the surfactant concentration increased, a reduction in particle size was observed. The design of experiment was exploited also by Zielinska et al. to develop citral-loaded SLN by hot high-pressure homogenization [195]. The 2^2^ factorial design determined the optimal concentration of components: 1 wt% of citral, 4 wt% of lipid glycerol monostearate and 2.5 wt% of poloxamer 188, selected as surfactant to obtain homogeneous nanoparticles with mean size lower than 100 nm. In the same study, the anti-inflammatory activity of citral and geraniol, comparing pure EOs and EO-loaded SNL, was evaluated. The analysis of the inhibition of NO production, measured on RAW 264.7 cells, showed a greater anti-inflammatory activity of citral compared to geraniol. The results of the cytotoxicity assay showed that cell viability in HaCaT cells was significantly reduced by citral, even if its effect was more evident in the treatment of A431 cells, thus demonstrating the skin anti-cancer potential of this monoterpene [195]. Saporito and co-workers recently developed lipid nanoparticles of first generation (SLN) and second generation (nanostructured lipid carriers, NLC) (Figure 6) for the delivery of eucalyptus or rosemary EOs as potential medical devices, to improve healing of skin wounds [196].

NLC represent a second generation of lipid nanoparticles, born in 1999 in order to overcome the limits related to SLN, such as limited drug-loading capacity and potential drug expulsion during storage. NLC are characterized by the presence of a liquid lipid, together with the solid lipid, which allows the formation of an imperfect or amorphous structure, able to guarantee higher drug loading values compared to SLN, thus avoiding drug loss during storage [185]. Cocoa butter was selected as solid lipid, while olive oil or sesame oil were used as liquid lipids in NLC formulations [196]. Interestingly, NLC showed higher bioadhesive properties compared to SLN, probably due to their flexible structure which would promote interactions with biologic substrate, thus promoting lesion closure. As reported in the in vitro cytotoxicity studies, the presence of olive oil increased the cell viability due to the high content of oleic acid, previously demonstrated to promote cell proliferation. Furthermore, in vivo studies on a rat burn model confirmed that NLC prepared with olive oil and loaded with *Eucalyptus* EO were able to promote the closure of the wound, providing good reepithelization and stratum corneum formation [196]. Cocoa butter and olive oil were also used as solid lipid and liquid lipid respectively to encapsulate cardamom EO, producing small NLC (<150 nm) with an encapsulation efficiency higher than 90%. Results obtained from in vitro release studies and DPPH scavenging activity assay demonstrated that the DDS structure is able to protect the EO—during the storage—and thus enhance its antioxidant property, also because of the presence of the two natural oils [197]. The suitability of the monoterpene carvacrol as a component of binary mixtures of NLC solid lipids was deeply analyzed by Galvao and co-workers [198]. The considered solid lipids were stearic acid, beeswax, and carnauba wax, which were analyzed both as pure components and in mixture with different ratio of carvacrol (10%, 25%, and 50% *w/w*). The obtained binary mixtures showed lower values of enthalpy and melting temperatures, highlighting the relevance of this active ingredient in the formulation of NLC. Moreover, carvacrol demonstrated to increase the loading capacity and the encapsulation efficiency of carnauba wax and beeswax—through the extension of the interlayer—when compared with the pure waxes [198]. A comparison between beeswax or carnauba wax was also made in order to select the optimal solid lipid component in the formulation of clove oil-loaded NLC. It resulted that the presence of carnauba wax led to the formation of NLC with higher particles size and PDI values compared to the ones produced with beeswax; furthermore, even better values were obtained by also introducing the liquid lipid crodamol, with the production of NLC that were stable even after storage. Additionally, beeswax-crodamol nanoparticles showed a higher clove encapsulation efficiency (66 ± 1%–63 ± 0%) when compared with carnauba wax-crodamol ones (60 ± 2%–58 ± 0%): again, the presence of the liquid lipid is decisive. In both situations, the formulations showed no decrease in encapsulated clove percentages after 30 days storage, demonstrating the great suitability of these component in the formulation of NLC for clove delivery [199]. The wound-healing application of thymol-loaded NLC was recently investigated [200]. Herein, Pivetta and co-workers prepared NLC exploiting the hydrating, cicatrizing, and anti-inflammatory properties of two natural lipids, Illipe butter and Calendula oil, to encapsulate thymol EO, whose anti-inflammatory activity could improve the wound healing [200]. Thymol-NLC, with mean particles size < 150 nm, narrow size distribution (PDI < 0.3), negative zeta potential (−12.5 mV), and high encapsulation efficiency (90%), were prepared by hot emulsion followed by sonication. NLC were able to reduce the cytotoxicity of the EO on a non-tumorigenic immortalized human keratinocytes cell line (HaCatT). Thymol-NLC hydrogel obtained using Carbopol gel, selected for its high biological compatibility, were able to provide a fast EO release and to maintain the EO release over time. Interestingly, thymol-NLC hydrogel showed higher anti-inflammatory activity compared to the free EO, as confirmed by in vitro studies. The results of the in vivo antipsoriatic activity test performed using a model of imiquimod psoriasis-like inflammation, enabled to observe that the mice treated with thymol-NLC hydrogel showed a delay in the development of the inflammation and that the severity of the inflammation was reduced compared to the negative control group [200]. A combined approach to the treatment of wound healing was recently proposed by Carbone et al., who prepared ferulic acid-loaded NLC using *Lavandula* EO or isopropyl myristate (IPM) as the liquid oil component [201]. NLC with homogeneous particles lower than 150 nm were obtained by the phase inversion temperature (PIT) method; the presence of *Lavandula* EO, instead of the liquid synthetic oil, increased the long-term stability of the suspension, probably due to the tendency of the particles to flocculate. Interestingly, the morphological study revealed that the choice of the liquid oil component affected the NLC structure, since *Lavandula*-NLC presented small spherically shaped particles with a type-II (amorphous) structure, while the presence of the synthetic oil induced the formation of a similar multiple-type-III NLC, with the presence of grape-like aggregates of very small oil nanocomponents outside the main nanoparticle [201]. The type-II structure of ferulic acid-NLC prepared with Lavandula EO also affected the cumulative amount of drug released, which was found to be lower compared to those of NLC prepared with the synthetic oil (46% and 62%, respectively). Interestingly, the type-II structure also improved the cytocompatibility on human fibroblasts and enhanced fibroblast migration promoting wound healing. Therefore, authors concluded that a potential combined protective effect of the antioxidant drug and Lavandula EO was achieved using NLC, in which the co-presence shows a synergistic effect in promoting cell migration. In order to reduce the volatilization and improve the bioavailability of linalool EO, NLC were prepared and optimized by high-pressure homogenization using glycerin monostearate as solid lipid and decanoyl/octanoyl-glycerides as liquid lipid [202]. The optimized formulation obtained using 2.5% *w/w* of each lipid in the surfactant mixture span80/tween80 (2.0 and 4.0% *w/w*, respectively) showed homogeneous spherical particles of about 50 nm able to provide a controlled EO release. In vivo pharmacokinetics studies confirmed that linalool-loaded NLC were able to significantly improve the EO absorption and bioavailability, as confirmed by the higher values of t_1/2_, t_max_ and C_max_ obtained for the loaded EO compared to the free linalool solution [202]. Peppermint essential oil was analyzed in order to explore its wound-healing and antibacterial activity, when encapsulated into NLC. Although the encapsulation efficiency of EO into NLC has been found to be very high (93.2 ± 1.2%), in vitro studies on various bacterial cell lines have shown that its antimicrobial activity is comparable to the pure essential oil one; in particular, pure EO MIC values were in the range 5.16 ± 1.48–20.00 ± 0.00, while EO-loaded NLC MIC values were between 5.10 ± 1.49 and 20.00 ± 0.00. The importance of the incorporation of this EO into DDS has been highlighted, instead, by in vivo studies on infected wound models: the wound-healing rate was accelerated due to the ability of the EO-NLC to decrease the bacterial count, in addition to the intrinsic anti-inflammatory property of the EO, which was also improved by the NLC structure [203]. The antimicrobial activity of menthol-loaded NLC, with mean size of 115 nm and high EO encapsulation efficiency (98.73%), was investigated by Piran et al. [204]. In particular, menthol-loaded NLC showed higher antibacterial activity against fungi and Gram-positive bacteria compared to the EO emulsion: NLC were able to inhibit the growth of *Staphylococcus aureus*, *Bacillus cereus*, *Escherichia coli,* and *C. albicans* at concentration of 125, 250, 500, and 78 µg/mL, respectively, while the corresponding MIC values for menthol emulsion were 1000, 2000, 2000, and 156 µg/mL. Piran et al., therefore concluded that menthol encapsulation in NLC allows decreasing the amount of the EO for preserving foodstuffs from microorganism growth and spoilage. The influence of the preparation method in determining the characteristics of EO-loaded NLC was investigated by Carbone et al., who investigated a lab-scale (phase inversion temperature method) and a scalable (high-pressure homogenization) production methods [47]. Authors demonstrated that the greater energy used in the second method allowed obtaining smaller particles and more stable colloidal NLC, using *Rosmarinus officinalis* L., *Lavandula x intermedia* “Sumian” and *Origanum vulgare* subsp. *hirtum* as liquid lipid. Interestingly, results of in vitro biological cell viability test on murine macrophage cell line (RAW 264.7) showed that the nanoencapsulation of the EO enhanced its biocompatibility, being *Lavandula* and *Rosmarinus* NLC the most biocompatible formulations up to a concentration of 0.1% (*v/v*). Furthermore, the nanoencapsulation did not reduce the intrinsic anti-inflammatory activity of the EO, which was found to decrease in the order *Lavandula* > *Rosmarinus* ≥ *Origanum* [47]. Therefore, it was demonstrated that EO can be used as both active ingredients and oily components of NLC, thus enhancing the biocompatibility and reducing the cytotoxicity of the pure oils. The topical application of gel vehicles containing *Rosmarinus*-loaded NLC was also investigated, confirming the potentiality of the EO encapsulation strategy in the treatment of cutaneous alterations involving loss of skin hydration and elasticity [205]. *Lavandula* and *Rosmarinus* were shown to be anti-proliferative agents with the potential to be used as co-adjuvants in combination with clotrimazole-loaded NLC in the treatment of topical candidiasis [206]. In this recent study, homogeneous small sized NLC (<100 nm) with long-term stability were successfully prepared using *Lavandula* and *Rosmarinus* as liquid lipid, and loaded with clotrimazole. Interestingly, EO encapsulation into NLC induced an increase in the antiproliferative activity of the selected oils on keratinocytes cell line originated from human skin (HaCaT) and human epidermoid carcinoma cell line (A431), thus suggesting a possible use of *Rosmarinus* and/or *Lavandula*-loaded NLC as coadjuvants in non-cancerous proliferative skin diseases. The results of the in vitro test against *Candida albicans*, *Candida krusei*, and *Candida parapsilosis*, confirmed that clotrimazole-loaded NLC containing *Lavandula* or *Rosmarinus* were able to improve the antifungal drug activity, thus representing a promising strategy to enhance the effectiveness of traditional antimycotic drug against non-tumoral proliferative dermal diseases [206]. Topical delivery of drugs using DDS was further explored by Miranda and co-workers, studying *Ridolfia segetum* (L.) Moris essential oil, which was encapsulated into homogeneous NLC with a mean size of 143 ± 5 nm. To increase biocompatibility and permeation, also EO-NLC hydrogel were prepared. Using dialysis membrane, it was analyzed the in vitro release of the EO from the nanoplatform, which resulted to be biphasic: at the beginning a low release was detected, which boosted at about 12 h. The in vivo permeation of both EO-NLC and EO-NLC hydrogel was studied using newborn pig epidermal membranes and it remarked that the hydrogel structure allowed a higher permeation than the simple NLC. The skin retention studies also supported the evidence that the hydrogel NLC formulation was able to provide a sustained release of the EO, acting as a reservoir [207]. As noted in many of the above papers, particles dimension and polydispersion index appear to be significant for the development of appropriate nanoplatform systems. Therefore, the preformulation study is often decisive. A full factorial design approach was developed by Vieira and co-workers, in order to select the optimal amount of surfactant and solid lipid (2^2^ factorial design) to prepare Sucupira Oil-Loaded NLC. It was obtained that the particle size decreased with higher surfactant concentration, while increased with growing concentrations of solid lipid; moreover, polydispersity index and zeta potential both increased with higher surfactant or solid lipid concentrations [208]. The preformulation study was decisive also in the evaluation of the feasibility of spray-drying method in the production of redispersable lipid systems, considering both SLN and NLC loaded with *Syzygium aromaticum* EO. It was developed a quality by design study considering four variables, analyzed at two levels: two of them were related to formulation composition—the presence (1%) or absence (0%) of the liquid lipid oleic acid and the cationic surfactant CTAB—while the other two were referred to spray drying conditions—the inlet drying temperature (60 °C and 80 °C) and the drying aids ratio in relation to the total formulation weight (1:1 and 2:1). Throughout this analysis, it was highlighted a probable interaction between the lipid CTAB—which has positive charge—and the gum Arabic—which has negative charge—as revealed by the increase in particle size and the inversion of the zeta potential. After redispersion of the powders, dynamic light scattering measurements showed no significant changes in particle size and PDI, demonstrating that spray drying method is adequate for this aim [209].

## 5. Authors Opinion and Future Perspectives

To the best of our knowledge, there is no evidence about the effectiveness of EOs as primary treatment in pharmacological treatments. It is worth to remind that unlike many plants, which are marketed as biochemical actives in humans, therefore classified as drugs, after proper scientific studies about effectiveness and safety, EOs are not subjected to the same studies about reproducible usefulness and safety. To date, in the U.S. Food and Drug Administration (FDA) classification, EOs are considered either cosmetics/food supplements or drugs, depending on their intended use, however their sales and uses are not regulated by FDA. Companies who manufacture or market EOs have a legal responsibility for ensuring their safety. Therefore, researchers and users should take into consideration the quality of the EOs based on the reputation of their sources. Nevertheless, the researchers’ interest in EOs applications is increasing due to the possibility to exploit them.

The need of EO encapsulation represents a valid strategy to reach their pharmaceutical application, which is limited by their many drawbacks. Herein, we described different potential applications of EOs in the pharmaceutical field, and we presented the results obtained by different researchers in the development of lipid-based DDS for EOs encapsulation, such as micro and nanoemulsions, liposomes, SLN and NLC. According to literature data, all strategies demonstrated a good ability in improving EOs stability and effectiveness, increasing their bioavailability compared to the pure compound. Some interesting recent applications are related to the combined encapsulation of the EO with a conventional synthetic drug, in order to improve the effectiveness, the biocompatibility, and reducing the resistant mechanisms. As discussed above, the selection of the quali-quantitative composition of the formulation and of the preparation method represent key parameters for obtaining a final formulation with the most appropriate properties for the desired pharmaceutical application. A direct comparison of the different lipid-based delivery systems studied is not easily achievable due to the substantial differences existing among them in terms of structure and behavior. In particular, in the design of a proper system for the delivery of a specific EO, different variables should be taken into consideration, such as the production method (use of heat or organic solvents), the selection of biocompatible and biodegradable raw materials, and the desired nanocarrier properties (i.e., mean size, stability, encapsulation efficiency and release profile).

However, thinking in terms of a near-future prospective for potential EOs application among the various colloidal lipid-based carriers analyzed in this review, it is the opinion of the authors that NLC have a greater possibility of being employed as a nanomedicine in the combined delivery of EOs and conventional drugs. In fact, the possibility of using EOs as intrinsic components of the lipid matrix in combination with solid lipids and surfactants approved by international commits for safety drugs and administration could represent the new frontier of EOs combined coadjuvant therapy in nanomedicine.

In our opinion, the efforts of researchers should be more focused on the development of patentable products by scalable production methods of interest for the pharmaceutical industry. In order to exploit EOs potential use as coadjuvant to traditional drugs and therapies, thanks to their many interesting biological activities, further investigations should be developed to describe their mechanisms of action and their eventual toxicological side effects. Therefore, more in vivo studies should be undertaken to provide reliable results of pharmaceutical interest, thus allowing the pharmaceutical application of newest EO-loaded delivery systems as anti-inflammatory, antimicrobial, antioxidant, antifungal coadjuvant approaches.

## Figures and Tables

**Table 1:** Advantages and drawbacks of different essential oils (EOs) extraction methods.

Methods		Advantages	Olumn Title Drawbacks
**Conventional Methods**	Hydrodistillation	EOs and water are easily separated by decantation.	Long extraction time; Chemical alteration due to the prolonged boiling; Loss of some polar compounds in the evaporated water [15].
	Entrainment by water steam	Less artefacts are generated; The extraction time is reduced [9].	Several hours of heating; Degradation of thermos labile compounds; Odor deterioration.
	Organic solvent extraction	Alterations and chemical artefacts are avoided.	The organic solvent can leave residues in the oil produced, compromising the safety of the product (not usable for pharmaceutics) [16].
	Cold pressing	Native properties (in terms of beneficial compounds) are preserved [17,18,19,20,21,22]; Low costs; No plant safety problems.	Nutraceutical content is lower compared with the oil obtained by solvent extraction; Pungent odors, due to the breakdown products of glucosinolates [19,23].
**Innovative Methods**	Supercritical fluid extraction	Higher quality of extracts, with better activities [24]; Relatively low temperatures [25]; Chemical inertness [25].	High costs (equipment and maintenance); Necessity of high CO_2_ purity [26]; Affinity of the supercritical CO_2_ to low-polar and non-polar compounds [26].
	Subcritical extraction liquids	The extraction time is reduced; No loss of volatile and thermolabile compounds; Low costs [27].	Less presence of monoterpene compounds that in hydrodistillated oil [28]; High amount of extractant requires [28].
	Ultrasound-assisted extraction	High efficiency [29]; Low temperature [29]; Reduced solvent consumption [30]; Less energy input [30].	Potential formation of free radicals during sonolysis of the solvent, with consequent degradation of labile compound by oxidation.
	Microwave-assisted extraction	Reduction of extraction time; Environmentally friendly [31]; Reduction of solvents; Fast and efficient extraction; Better sensory properties.	Use of high temperatures with formation of undesirable compounds; Frequent use of toxic organic solvents [26].
	Instant controlled pressure drop	Reduction of extraction time; Decrease of energy and water consumption [32].	No significant disadvantages: it is currently considered the most efficient method of extracting essential oils [33].

**Table 2:** EOs generally recognized as safe by Food and Drug Administration (FDA). Source: https://www.ecfr.gov/cgi-bin/text-idx?SID=69c1693f1fe5cddde23bdc34d0731b05&mc=true&node=pt21.6.582&rgn=div5#se21.6.582_120 (accessed on 10 February 2021).

Common Name	Botanical Name of Plant Source
Alfalfa	*Medicago sativa* L.
Allspice	*Pimenta officinalis* Lindl.
Almond, bitter (free from prussic acid)	*Prunus amygdalus* Batsch, *Prunus armeniaca* L. or *Prunus persica* (L.) Batsch.
Ambrette (seed)	*Hibiscus moschatus* Moench.
Angelica root	*Angelica archangelica* L.
Angelica seed	Do.
Angelica stem	Do.
Angostura (cusparia bark)	*Galipea officinalis* Hancock.
Anise	*Pimpinella anisum* L.
Asafetida	*Ferula assa-foetida* L. and related spp. of Ferula.
Balm (lemon balm)	*Melissa officinalis* L.
Balsam of Peru	*Myroxylon pereirae* Klotzsch.
Basil	*Ocimum basilicum* L.
Bay leaves	*Laurus nobilis* L.
Bay (myrcia oil)	*Pimenta racemosa* (Mill.) J. W. Moore.
Bergamot (bergamot orange)	*Citrus aurantium* L. subsp. bergamia Wright et Arn.
Bitter almond (free from prussic acid)	*Prunus amygdalus* Batsch, *Prunus armeniaca* L., or *Prunus persica* (L.) Batsch.
Bois de rose	*Aniba rosaeodora* Ducke.
Cacao	*Theobroma cacao* L.
Camomile (chamomile) flowers, Hungarian	*Matricaria chamomilla* L.
Camomile (chamomile) flowers, Roman or English	*Anthemis nobilis* L.
Cananga	*Cananga odorata* Hook. f. and Thoms.
Capsicum	*Capsicum frutescens* L. and *Capsicum annuum* L.
Caraway	*Carum carvi* L.
Cardamom seed (cardamon)	*Elettaria cardamomum* Maton.
Carob bean	*Ceratonia siliqua* L.
Carrot	*Daucus carota* L.
Cascarilla bark	*Croton eluteria* Benn.
Cassia bark, Chinese	*Cinnamomum cassia* Blume.
Cassia bark, Padang or Batavia	*Cinnamomum burmanni* Blume.
Cassia bark, Saigon	*Cinnamomum loureirii* Nees.
Celery seed	*Apium graveolens* L.
Cherry, wild, bark	*Prunus serotina* Ehrh.
Chervil	*Anthriscus cerefolium* (L.) Hoffm.
Chicory	*Cichorium intybus* L.
Cinnamon bark, Ceylon	*Cinnamomum zeylanicum* Nees.
Cinnamon bark, Chinese	*Cinnamomum cassia* Blume.
Cinnamon bark, Saigon	*Cinnamomum loureirii* Nees.
Cinnamon leaf, Ceylon	*Cinnamomum zeylanicum* Nees.
Cinnamon leaf, Chinese	*Cinnamomum cassia* Blume.
Cinnamon leaf, Saigon	*Cinnamomum loureirii* Nees.
Citronella	*Cymbopogon nardus* Rendle.
Citrus peels	*Citrus* spp.
Clary (clary sage)	*Salvia sclarea* L.
Clove bud	*Eugenia caryophyllata* Thunb.
Clove leaf	Do.
Clove stem	Do.
Clover	*Trifolium* spp.
Coca (decocainized)	*Erythroxylum coca* Lam. and other spp. of Erythroxylum.
Coffee	*Coffea* spp.
Cola nut	*Cola acuminata* Schott and Endl., and other spp. of Cola.
Coriander	*Coriandrum sativum* L.
Corn silk	*Zea mays* L.
Cumin (cummin)	*Cuminum cyminum* L.
Curacao orange peel (orange, bitter peel)	*Citrus aurantium* L.
Cusparia bark	*Galipea officinalis* Hancock.
Dandelion	*Taraxacum officinale* Weber and *T. laevigatum* DC.
Dandelion root	Do.
Dill	*Anethum graveolens* L.
Dog grass (quackgrass, triticum)	*Agropyron repens* (L.) Beauv.
Elder flowers	*Sambucus canadensis* L. and *S. nigra* L.
Estragole (esdragol, esdragon, tarragon)	*Artemisia dracunculus* L.
Estragon (tarragon)	Do.
Fennel, sweet	*Foeniculum vulgare* Mill.
Fenugreek	*Trigonella foenum-graecum* L.
Galanga (galangal)	*Alpinia officinarum* Hance.
Garlic	*Allium sativum* L.
Geranium	*Pelargonium* spp.
Geranium, East Indian	*Cymbopogon martini* Stapf.
Geranium, rose	*Pelargonium graveolens* L’Her.
Ginger	*Zingiber officinale* Rosc.
Glycyrrhiza	*Glycyrrhiza glabra* L. and other spp. of Glycyrrhiza.
Glycyrrhizin, ammoniated	Do.
Grapefruit	*Citrus paradisi* Macf.
Guava	*Psidium* spp.
Hickory bark	*Carya* spp.
Horehound (hoarhound)	*Marrubium vulgare* L.
Hops	*Humulus lupulus* L.
Horsemint	*Monarda punctata* L.
Hyssop	*Hyssopus officinalis* L.
Immortelle	*Helichrysum augustifolium* DC.
Jasmine	*Jaminum officinale* L. and other spp. of Jasminum.
Juniper (berries)	*Juniperus communis* L.
Kola nut	*Cola acuminata* Schott and Endl., and other spp. of Cola.
Laurel berries	*Laurus nobilis* L.
Laurel leaves	*Laurus* spp.
Lavender	*Lavandula officinalis* Chaix.
Lavender, spike	*Lavandula latifolia* Vill.
Lavandin	Hybrids between *Lavandula officinalis* Chaix and *Lavandula latifolin* Vill.
Lemon	*Citrus limon* (L.) Burm. f.
Lemon balm (see balm).	
Lemon grass	*Cymbopogon citratus* DC. and *Cymbopogon flexuosus* Stapf.
Lemon peel	*Citrus limon* (L.) Burm. f.
Licorice	*Glycyrrhiza glabra* L. and other spp. of Glycyrrhiza.
Lime	*Citrus aurantifolia* Swingle.
Linden flowers	*Tilia* spp.
Locust bean	*Ceratonia siliqua* L.
Lupulin	*Humulus lupulus* L.
Mace	*Myristica fragrans* Houtt.
Malt (extract)	*Hordeum vulgare* L., or other grains.
Mandarin	*Citrus reticulata* Blanco.
Marjoram, sweet	*Majorana hortensis* Moench.
Mate 1	*Ilex paraguariensis* St. Hil.
Melissa (see balm).	
Menthol	*Mentha* spp.
Menthyl acetate	Do.
Molasses (extract)	*Saccharum officinarum* L.
Mustard	*Brassica* spp.
Naringin	*Citrus paradisi* Macf.
Neroli, bigarade	*Citrus aurantium* L.
Nutmeg	*Myristica fragrans* Houtt.
Onion	*Allium cepa* L.
Orange, bitter, flowers	*Citrus aurantium* L.
Orange, bitter, peel	Do.
Orange leaf	*Citrus sinensis* (L.) Osbeck.
Orange, sweet	Do.
Orange, sweet, flowers	Do.
Orange, sweet, peel	Do.
Origanum	*Origanum* spp.
Palmarosa	*Cymbopogon martini* Stapf.
Paprika	*Capsicum annuum* L.
Parsley	*Petroselinum crispum* (Mill.) Mansf.
Pepper, black	*Piper nigrum* L.
Pepper, white	*Piper nigrum* L.
Peppermint	*Mentha piperita* L.
Peruvian balsam	*Myroxylon pereirae* Klotzsch.
Petitgrain	*Citrus aurantium* L.
Petitgrain lemon	*Citrus limon* (L.) Burm. f.
Petitgrain mandarin or tangerine	*Citrus reticulata* Blanco.
Pimenta	*Pimenta officinalis* Lindl.
Pimenta leaf	*Primenta officinalis* Lindl.
Pipsissewa leaves	*Chimaphila umbellata* Nutt.
Pomegranate	*Punica granatum* L.
Prickly ash bark	*Xanthoxylum* (or *Zanthoxylum*) *Americanum* Mill. or *Xanthoxylum clava-herculis* L.
Rose absolute	*Rosa alba* L., *Rosa centifolia* L., *Rosa damascena* Mill., *Rosa gallica* L., and vars. of these spp.
Rose (otto of roses, attar of roses)	Do.
Rose buds	Do.
Rose flowers	Do.
Rose fruit (hips)	Do.
Rose geranium	*Pelargonium graveolens* L’Her.
Rose leaves	*Rosa* spp.
Rosemary	*Rosmarinus officinalis* L.
Rue	*Ruta graveolens* L.
Saffron	*Crocus sativus* L.
Sage	*Salvia officinalis* L.
Sage, Greek	*Salvia triloba* L.
Sage, Spanish	*Salvia lavandulaefolia* Vahl.
St. John’s bread	*Ceratonia siliqua* L.
Savory, summer	*Satureia hortensis* L.
Savory, winter	*Satureia montana* L.
Schinus molle	*Schinus molle* L.
Sloe berries (blackthorn berries)	*Prunus spinosa* L.
Spearmint	*Mentha spicata* L.
Spike lavender	*Lavandula latifolia* Vill.
Tamarind	*Tamarindus indica* L.
Tangerine	*Citrus reticulata* Blanco.
Tannic acid	Nutgalls of *Quercus infectoria* Oliver and related spp. of Quercus. Also in many other plants.
Tarragon	*Artemisia dracunculus* L.
Tea	*Thea sinensis* L.
Thyme	*Thymus vulgaris* L. and *Thymus zygis* var. gracilis Boiss.
Thyme, white	Do.
Thyme, wild or creeping	*Thymus serpyllum* L.
Triticum (see dog grass).	
Tuberose	*Polianthes tuberosa* L.
Turmeric	*Curcuma longa* L.
Vanilla	*Vanilla planifolia* Andr. or *Vanilla tahitensis* J. W. Moore.
Violet flowers	*Viola odorata* L.
Violet leaves	Do.
Violet leaves absolute	Do.
Wild cherry bark	*Prunus serotina* Ehrh.
Ylang-ylang	*Cananga odorata* Hook. f. and Thoms.
Zedoary bark	*Curcuma zedoaria* Rosc.
